# Impairment of Oligodendroglia Maturation Leads to Aberrantly Increased Cortical Glutamate and Anxiety-Like Behaviors in Juvenile Mice

**DOI:** 10.3389/fncel.2015.00467

**Published:** 2015-12-15

**Authors:** Xianjun Chen, Weiguo Zhang, Tao Li, Yu Guo, Yanping Tian, Fei Wang, Shubao Liu, Hai-Ying Shen, Yue Feng, Lan Xiao

**Affiliations:** ^1^Department of Histology and Embryology, Chongqing Key Laboratory of Neurobiology, Third Military Medical UniversityChongqing, China; ^2^Department of Radiology, Institute of Surgery Research, Daping Hospital, Third Military Medical UniversityChongqing, China; ^3^Robert Stone Dow Neurobiology Laboratories, Legacy Research Institute, PortlandOR, USA; ^4^Department of Pharmacology, Emory University School of Medicine, AtlantaGA, USA

**Keywords:** oligodendrocyte, brain development, *Olig2* knockout, cortical neurons, glutamate, anxiety behavior

## Abstract

Adolescence is the critical time for developing proper oligodendrocyte (OL)-neuron interaction and the peak of onset for many cognitive diseases, among which anxiety disorders display the highest prevalence. However, whether impairment of *de novo* OL development causes neuronal abnormalities and contributes to the early onset of anxiety phenotype in childhood still remains unexplored. In this study, we tested the hypothesis that defects in OL maturation manifests cortical neuron function and leads to anxiety-like behaviors in juvenile mice. We report here that conditional knockout of the *Olig2 gene* (*Olig2* cKO) specifically in differentiating OLs in the mouse brain preferentially impaired OL maturation in the gray matter of cerebral cortex. Interestingly, localized proton magnetic resonance spectroscopy *revealed that Olig2* cKO mice displayed abnormally elevated cortical glutamate levels. In addition, transmission electron microscopy demonstrated increased vesicle density in excitatory glutamatergic synapses in the cortex of the *Olig2* cKO mice. Moreover, juvenile *Olig2* cKO mice exhibited anxiety-like behaviors and impairment in behavioral inhibition. Taken together, our results suggest that impaired OL development affects glutamatergic neuron function in the cortex and causes anxiety-related behaviors in juvenile mice. These discoveries raise an intriguing possibility that OL defects may be a contributing mechanism for the onset of anxiety in childhood.

## Introduction

Adolescence is a peak time for the onset of numerous mental disorders, represented by anxiety, impulse control disorders, and schizophrenia (Paus et al., 2008). One in five children and adolescents suffers from a mental illness that persists into adulthood. Among the numerous mental disorders, the prevalence of anxiety is the highest (Kessler et al., 2005). Consistent with the emotionality, risk-taking and impulsivity characters (Butters, 2011), anxiety phenotypes often begin in childhood and early adolescence, sometimes continue into the adulthood (Lee et al., 2014). However, molecular and cellular mechanisms that lead to anxiety in adolescence remain unknown, which is a prevailing issue in understanding the early onset of mental dysfunction.

In the adolescent brain, the structure of neuronal circuits and the functional properties of neurons are highly plastic (Tau and Peterson, 2010). Not only the number and morphology of synapses are dynamically altered (Penzes et al., 2011), the availability of neurotransmitters and corresponding receptors also undergo differential regulation in various brain regions (Lee et al., 2014). Conceivably, a transient interference of neurotransmission may markedly affect the functional balance of neuronal circuitry in the young brain, thus resulting in dysregulated emotions and actions (Casey et al., 2008). Moreover, recent clinical studies revealed that aberrantly elevated glutamate levels in the anterior cingulate cortex were positively correlated with clinical symptoms of anxiety and impulsivity in patients (Phan et al., 2005; Hoerst et al., 2010; Modi et al., 2014). These findings suggest that abnormal cortical glutamate homeostasis may contribute to the pathogenesis of anxiety disorders, while the underlying mechanisms remain undefined.

Synaptic transmission and cognitive ability are tightly regulated by neuron-glia interaction (de Hoz and Simons, 2015). Emerging evidence suggests key roles of oligodendroglia (OL) in brain function and psychiatric diseases (Fields, 2008). In particular, the clinical onset of anxiety peaks during vigorous OL and myelin development in the cerebral cortex (Kessler et al., 2005; Miller et al., 2012; Young et al., 2013). Besides the classical view of OL function in myelination that is essential for saltatory conductance, recent studies revealed that OL also provides trophic factors and metabolites that are critical for modulating neuronal function and plasticity (Du and Dreyfus, 2002; Funfschilling et al., 2012; Lee et al., 2012). In fact, essential role of OL is demonstrated in higher brain function, including learning motor skills, long distance connectivity and spiking-timing-dependent plasticity (McKenzie et al., 2014; Nave and Ehrenreich, 2014). Moreover, OL impairment is frequently observed in neuropsychiatric disorders, including schizophrenia, bipolar, and major depression (Fields, 2008; Edgar and Sibille, 2012; Cassoli et al., 2015). Nonetheless, whether defects in OL development during childhood and early adolescence may affect cortical neurons and contribute to the anxiety behaviors still remain unexplored.

In this study, we explored whether specific impairment of *de novo* OL maturation may affect cortical glutamate and result in maladaptive behaviors in juvenile mice. The *Olig2* gene encodes a transcription factor essential for OL lineage specification, differentiation and myelination (Zhou et al., 2000; Lu et al., 2002; Ligon et al., 2006; Yue et al., 2006; Mei et al., 2013). We showed that conditional deletion of the *Olig2* gene specifically in differentiated OLs resulted in preferential reduction of mature OL in the cerebral cortex. Interestingly, *Olig2* cKO mice display abnormally elevated glutamate levels in the cortex and increased density of vesicles in cortical glutamatergic synapses in young mice. Moreover, juvenile *Olig2* cKO mice showed anxiety and impulsivity-like behaviors. Taking together, our results indicate that impaired OL development in the cortex interferes with glutamate function and lead to anxiety- and impulsivity-like behaviors reminiscent of adolescent mental illnesses.

## Materials and Methods

### Animals

The *Olig2*-flox mice were previously described (Yue et al., 2006) in which the *Olig2* coding region is flanked by two inserted loxp sites. The mice that express Cre recombinase under the control of the *CNPase* (*CNP*) promoter (*CNP-Cre* mice) were also previously described (Lappe-Siefke et al., 2003) where *Cre* recombinase was knocked into one allele of the *CNP* gene locus. To delete *Olig2* in oligodendroglia lineage cells, *Olig2^loxp/loxp^* mice were crossed with the *CNP-Cre^+/-^* mice to generate both *CNP-Cre^+^*; *Olig2^loxp/loxp^* offspring (*Olig2* cKO) and *CNP-Cre^-^; Olig2^loxp/loxp^* littermates (WT).

### Ethics Statement

All animal experiments were performed according to an approved protocol from the Laboratory Animal Welfare and Ethics Committee of the Third Military Medical University.

### Immunohistochemistry

At the age of postnatal day 21 (P21), mice (*n* = 6 for each group) were deeply anesthetized with 1% sodium pentobarbital and transcardially perfused with 4% paraformaldehyde in PBS. Brains were dehydrated in 10, 20, 30% sucrose in 4% paraformaldehyde for 12 h, respectively. The frozen brains were sectioned (20 μm) on a cryostat microtome (MS 1900, Leica). Free-floating sections were incubated with primary antibodies overnight at 4°C after blocking with PBS containing 0.3% TritonX-100 and 5% bovine serum at 37°C for 1 h. Then they were incubated with secondary antibodies for 3 h following by SABC regent (1:200; VECTASTAIN) for 1h at room temperature. The antigen-antibody complexes were visualized using DAB (Boster) as the chromogen. The primary antibodies included: mouse anti-CC1 (1:500; Millipore), rabbit anti-PDGFRα (1:200; Santa Cruz Biotechnology). Biotinylated secondary antibodies to rabbit (1:1000; VECTOR BA1000) or mouse (1:1000; VECTOR BA9200) were used as indicated in the legends.

### Western Blotting

At the age of P21, mice (*n* = 7 in WT, *n* = 8 in *Olig2* cKO) were anesthetized with 1% pentobarbital. Cerebral cortex (anterior cingulated area) and corpus callosum were rapidly removed and frozen. Frozen samples were homogenized and proteins were extracted using RIPA lyses buffer with protease inhibitors (Roche). Lysates containing 40 μg protein were denatured in gel-loading buffer, separated on 10% SDS-PAGE gels, transferred to PVDF membranes and visualized by chemiluminescence (ECL plus, GE Healthcare). Quantification of band intensity was analyzed using Image-Pro Plus software 5.0 (Media Cybernetics). The following primary antibodies were used: mouse anti-Olig2 (1:500; Millipore), mouse anti-MBP (1:1000; Santa Cruz Biotechnology) and mouse anti-β-actin (1:2000; Santa Cruz Biotechnology).

### MRI and MRS

At the age of P21, MRI and MRS were performed, as recently described (Michaelis et al., 2009), on *Olig2* cKO (*n* = 7) and WT control (*n* = 6). Mice were initially anesthetized with 5% isoflurane, subsequently incubated and kept under anesthesia with 1.75% isoflurane in ambient air. *In vivo* localized proton MRS (PRESS, TR/TE = 3,000/20 ms) in cerebral cortex (2.5 mm × 1 mm × 2.5 mm) was performed at 7.0T (Bruker BioSpec 70/20 USR). T2-weighted MRI (Turbo-RARE, TR/TE = 1,500/35ms, 8 echoes, slice thickness 500 μm) in axial and sagittal orientation served to ensure a proper position of volumes-of-interest. Metabolite quantification involved spectral evaluation by LCModel and calibration with brain water concentration (Provencher, 1993). Metabolites with Cramer-Rao lower bounds above 20% were excluded from further analyses.

### Quantification of Glutamate in Cerebral Cortex

At the age of P21, mice (n = 6 for each group) were initially anesthetized with 5% isoflurane. Cerebral cortex (anterior cingulate area) was removed and frozen at -80°C immediately. The brain tissues were homogenized with 100 μl glutamate assay buffer and followed the manufacturer’s instruction from Glutamate assay kit (Sigma). The 10KD spin filters (Biovision) were applied. The absorbance was measured at 450 nm with Model 680 microplate reader (Bio-Rad).

### Electron Microscopy

Electron microscopy (EM) analysis was performed as previously described (Mei et al., 2013). At the age of P21, mice were initially anesthetized with 5% isoflurane. The anterior cingulate cortex of *Olig2* cKO mice (*n* = 3) and WT controls (*n* = 3) were removed rapidly and fixed in fresh fixative overnight at 4°C. Tissue cubes (1 mm × 1 mm × 1 mm) were rinsed in PBS, postfixed in 1% OsO4 in PBS for 2 h, counterstained with uranyl acetate, dehydrated in a graded ethanol series, infiltrated with propylene oxide, and embedded in Epon. Ultrathin sections (∼60 nm) were generated by an ultramicrotome (LKB-V, LKB Produkter AB, Bromma) and were viewed with a transmission electron microscope (TECNAI10, Philips). Three sections from each mouse were investigated at a comparable location from *Olig2* cKO and WT control mice under magnification of 60k. Digital images were acquired with an AMT XR-60 CCD Digital Camera System and analyzed using Image-Pro Plus software 5.0 (Media Cybernetics).

### Behavioral Tests

*Olig2* cKO mice and WT littermates were housed in a controlled environment (25°C) with free access to food and water and maintained on a 12 h/12 h day/night cycle. Behavioral tests were conducted on sex-balanced groups of experimentally naive mice at P21. All tests were done from 10:00 h to 18:00 h. One group of mice were tested in open-field test (between 10:00 h and 12:00 h) and elevated plus-maze test (between 15:00 h and 18:00 h) on the same day to measure anxiety. Another group of mice were only tested in cliff avoidance reaction (CAR) test (between 10:00 h and 18:00 h) to measure impulsivity. After each experiment, all the apparatuses were wiped clean with 70% ethanol to prevent a bias due to olfactory cues. For all behavioral experiments, investigators were blinded for genotype and mice were gently handled to avoid stress.

Open field test was performed using an open-field activity system (Biowill, Shanghai, China), as described (Wang et al., 2013). Briefly, mice (*n* = 26 in WT, *n* = 18 in *Olig2* cKO) were placed in the center of the open-field box (50 cm × 50 cm × 50 cm), and activity was recorded during a period of 10 min. The total and center-area travel distances were measured and the time spent in the central area was recorded.

Elevated plus-maze test was conducted as previously described (Katayama et al., 2010). Briefly, mice (*n* = 24 in WT, *n* = 15 in *Olig2* cKO) were tested on a plus-maze apparatus (Biowill, Shanghai, China) [closed arms, 25 × 5 × 15 (H) cm; open arms 25 × 5 × 0.3 (H) cm] arranged orthogonally 60 cm above the floor. Each mouse was initially placed in the center area facing an open arm, and then allowed to move freely in the maze for 5 min. The total travel distance on the maze, time spent on any arms and entries into any arms were recorded.

Cliff avoidance reaction test was conducted as previously described with slight modification (Yamashita et al., 2013). Mice (*n* = 14 in WT, *n* = 15 in *Olig2* cKO) were assessed using a round platform (diameter, 16 cm; height, 50 cm). The test was initiated by gently placing an animal on the platform with the forelimbs approached the edge. If the animal fell from the platform during the 20 min test, it was judged to have impaired CAR. The latency from an initial placement on the platform until falling was recorded. The incidence of impaired CAR was calculated as a percentage index for each group: % (CAR) = (the number of intact CAR mice (which did not fall from platform)/total number of tested mice) × 100. Duration of each entry into the edge area, which was defined as an outer ring with a width of 2 cm, was recorded before the mouse fell off.

### Statistical Analysis

We performed statistical analyses using the SPSS software version 13.0. All the data were confirmed to be normal distribution, as tested by the Kolmogorov–Smirnov test. For between-group comparisons, we used the Independent-Samples *t*-test with Welch correction, if the variance was unequal. We considered results to be significant at *p* < 0.05.

## Results

### The Loss of *Olig2* Preferentially Impaired OL Maturation in the Cerebral Cortex

**FIGURE 1:**
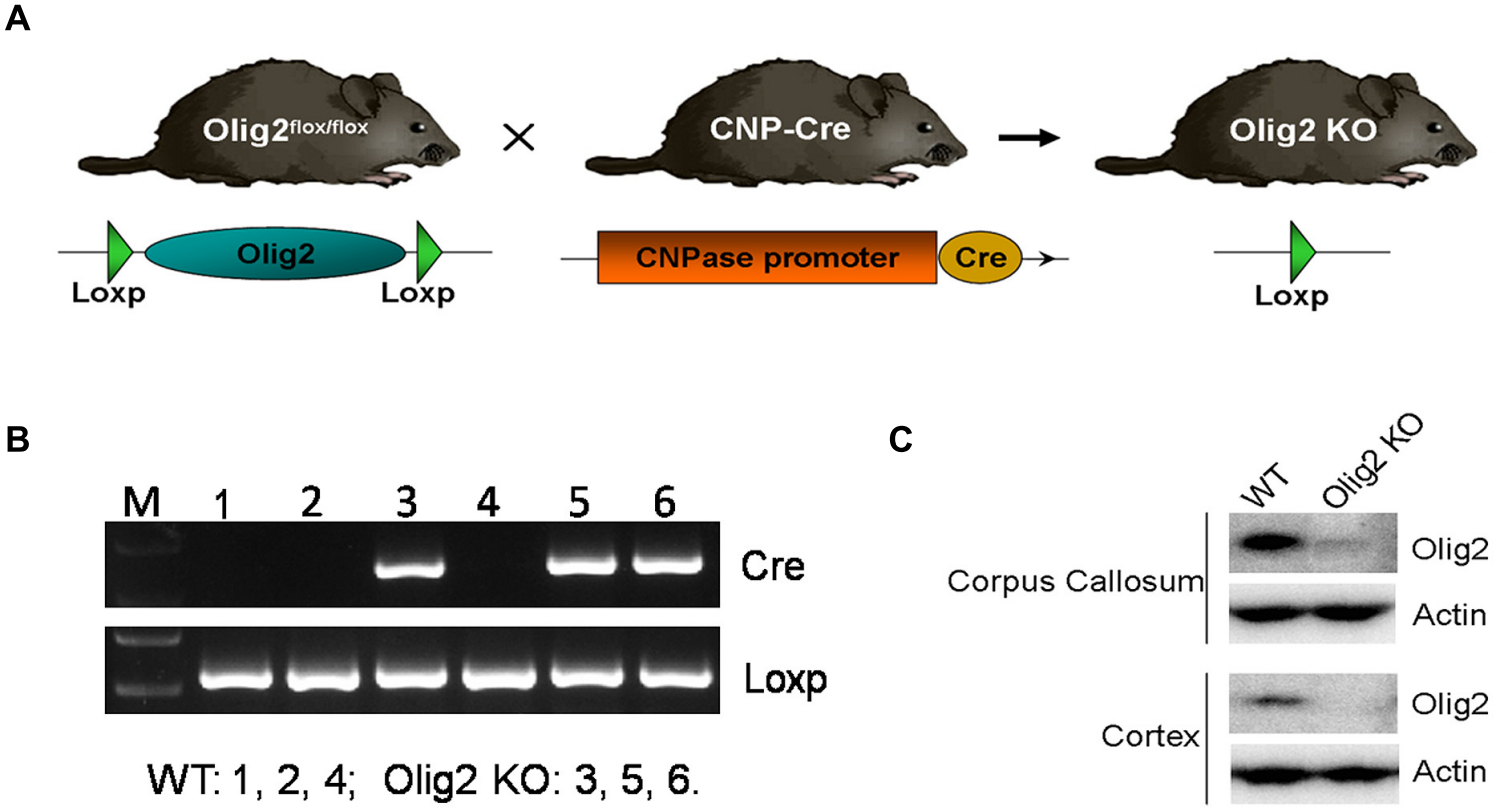
**Generating *Olig2* conditional knockout mice. (A)** Diagram shows *Olig2^flox/flox^* mice were crossed with mice that express *Cre* recombinase under the control of the *CNPase* promoter to generate *Olig2* cKO mice. **(B)** The offspring were genotyped by PCR for cnp-cre and floxed Olig2 alleles. **(C)** Western Blot analyses showed an absence of Olig2 protein in cortex and corpus callosum of *Olig2* cKO mice.

**FIGURE 2:**
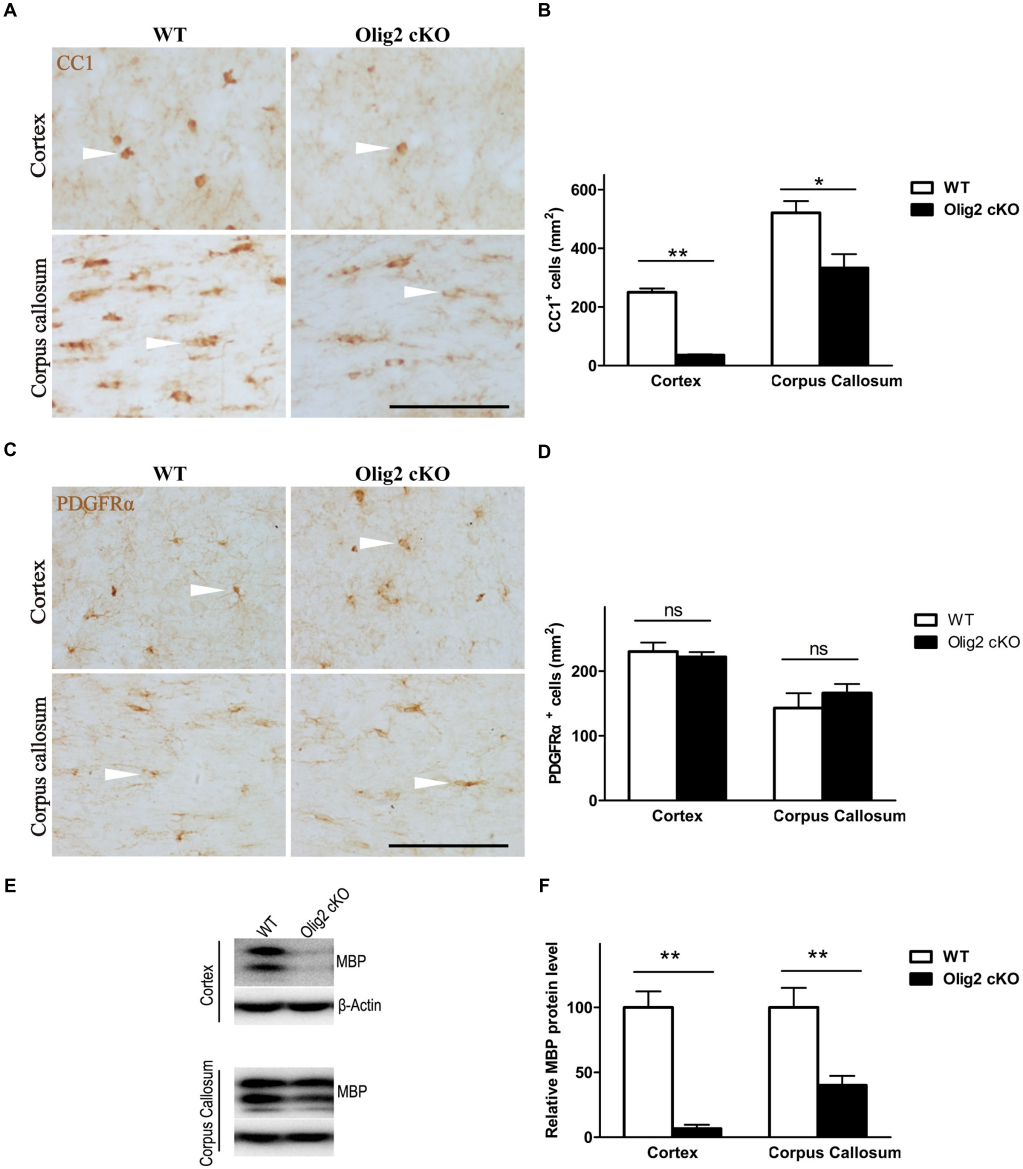
**Preferential impairment of oligodendroglia maturation in the cortex of *Olig2* cKO mice. (A)** Representative images of immunohistochemistry of CC1 showing mature oligodendrocytes (OLs) (white arrow head) in the cortex and corpus callosum from WT and *Olig2* cKO mice. **(B)** Quantification of the density of CC1^+^ cells in the cortex and corpus callosum from WT and *Olig2* cKO mice. **(C)** Representative images of immunohistochemistry of PDGFRα^+^ showing oligodendroglia progenitor cells (OPCs) in the cortex and corpus callosum from WT and *Olig2* cKO mice. **(D)** Quantification of the density of PDGFRα^+^ cells in the cortex and corpus callosum from WT and *Olig2* cKO mice. **(E)** Representative Western blot of MBP expression in the cortex and corpus callosum from WT or *Olig2* cKO mice. **(F)** Quantification of MBP signal on the immunoblot of the cortex and corpus callosum from WT or *Olig2* cKO mice normalized to β-actin as a loading control. Scale bars = 100 μm. Data are presented as mean ± SEM. ns, no significant different; ^∗^*P* < 0.05; ^∗∗^*P* < 0.01.

To explore the consequence of impaired OL development on brain function, conditional deletion of the *Olig2* gene was achieved in differentiated OL during neonatal development by introducing expression of the cre recombinase under transcriptional control of the *CNPase* promoter (*CNP-Cre*) in the *Olig2-loxp* mice (**Figure 1A**). The offspring were genotyped by PCR at postnatal day 7 (P7) to identify *Olig2^loxp/loxp^* mice that carry the *CNP-Cre* allele (*Olig2* cKO) and *Olig2^loxp/loxp^* littermates that lack the *CNP-Cre* allele (WT control) (**Figure 1B**). The knockout efficiency of *Olig2* was confirmed by the diminished expression of the *Olig2* protein in both the corpus callosum and cerebral cortex of the *Olig2* cKO mice at P21 (**Figure 1C**). To investigate the effects of *Olig2* loss on OL development, we quantified oligodendroglia progenitor cells (OPCs) marked by immunohistochemistry staining of PDGF receptor α (PDGFRα) and mature OL marked by CC1. As shown in **Figure 2**, the numbers of CC1^+^ mature OL in *Olig2* cKO mice were significantly decreased by 85.6% in the cortex (250 ± 13 in WT vs. 36 ± 2.8 in *Olig2* cKO) and 36.1% (521 ± 40 in WT vs. 333 ± 47 in *Olig2* cKO) in the corpus callosum, respectively (**Figures 2A,B**). In contrast, the density of PDGFRα^+^ OPCs was not affected (**Figures 2C,D**). These results suggest that OL maturation is preferentially affected in the cerebral cortex of the *Olig2* cKO. Such a conclusion is further supported by the observation that myelin basic protein (MBP) was reduced 94% in the *Olig2* cKO cortex (100 ± 12.3% in WT vs. 6.6 ± 3.0% in *Olig2* cKO), but only reduced 60% in the *Olig2* cKO corpus callosum (100 ± 15.1% in WT vs. 40.0 ± 7.3% in *Olig2* cKO) (**Figures 2E,F**).

### The Cerebral Cortex of *Olig2* cKO Mice Harbored Abnormally Higher Levels of Glutamate

**FIGURE 3:**
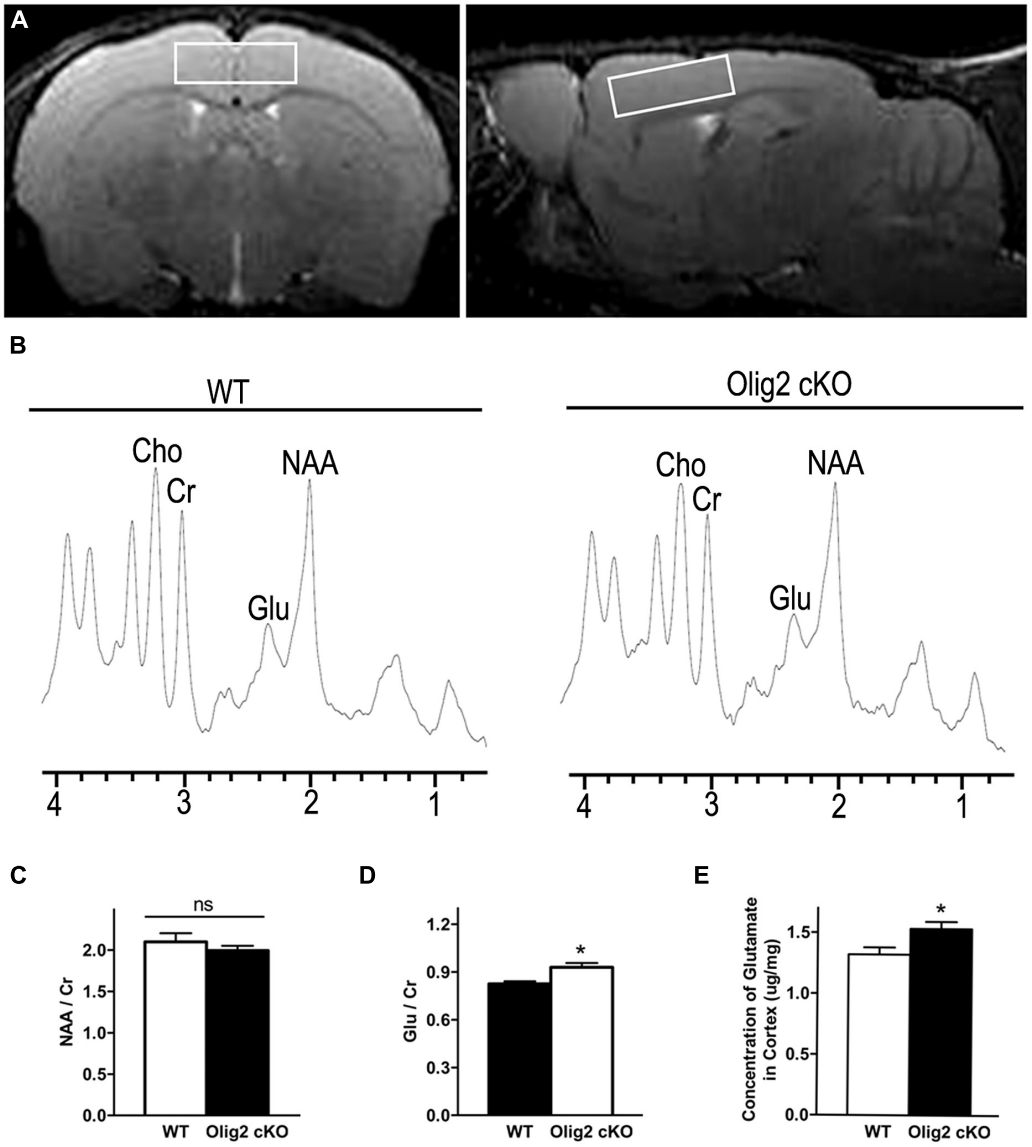
**Aberrantly increased glutamate level in the cortex of *Olig2* cKO mice. (A)** Representative axial and sagittal T2-weighted MR images indicate the position and orientation of point-resolved spectroscopy voxel in the cortex (white rectangle). **(B)** Localized proton magnetic resonance spectra of the cortex from WT or *Olig2* cKO mice, respectively. NAA, *N*-acetylaspartate; Glu, Glutamate; Cr, total creatine; ppm, parts per million. **(C)** Quantification of the ratio of NAA/Cr in the cortex of WT or *Olig2* cKO mice. **(D)** Quantification of the ratio of Glu/Cr in the cortex of WT or *Olig2* cKO mice. **(E)** Quantification of glutamate concentration in the cortex by the enzymatic assay from WT or *Olig2* cKO mice. Data are mean ± SEM. ns, no significant difference. ^∗^*P* < 0.05.

To explore whether and how impaired OL maturation may interfere with neuronal function in the juvenile brain, we examined the spectrum of neurochemicals in the cortex of WT and *Olig2* cKO mice at the age of P21 using proton magnetic resonance spectroscopy (1H MRS). T2-weighted MRI images in axial and sagittal orientation were used to ensure the volume-of-interest (**Figure 3A**). Representative MRS spectrums showed resonance of various neurochemicals (**Figure 3B**). *N*-acetylaspartate (NAA) and glutamate (Glu) are the two most abundant neurochemicals in the brain. The peak areas for NAA and Glu were normalized to that of creatine (Cr) for quantitative comparison between the genotypes. Interestingly, the *Olig2* cKO cortex harbors a moderate yet statistically significant increase of Glu/Cr than WT littermates (0.83 ± 0.02 in WT vs. 0.93 ± 0.03 in *Olig2* cKO, *P* = 0.01) (**Figure 3D**). In contrast, the ratio of NAA/Cr, which is thought to reflect neuronal health or viability (Urenjak et al., 1993), was not altered in the *Olig2* cKO mutant (*P* = 0.379) (**Figure 3C**). In addition, choline (Cho) level showed a trend of decrease in the *Olig2* cKO cortex, although no statistical significance was achieved (data not shown). The increase of glutamate in *Olig2* cKO cortex was further validated by enzymatic assay, in which a significant increase of total glutamate in the cortex of *Olig2* cKO mice was observed (1.32 ± 0.06 μg/mg in WT vs. 1.53 ± 0.06 μg/mg in *Olig2* cKO, *P* = 0.029) (**Figure 3E**). These results demonstrated that impaired OL maturation specifically leads to an abnormal increase of glutamate in the cortex without altering neuronal viability.

### Glutamatergic Synapses of *Olig2* cKO Cortical Neurons Contained Higher Density of Synaptic Vehicles

**FIGURE 4:**
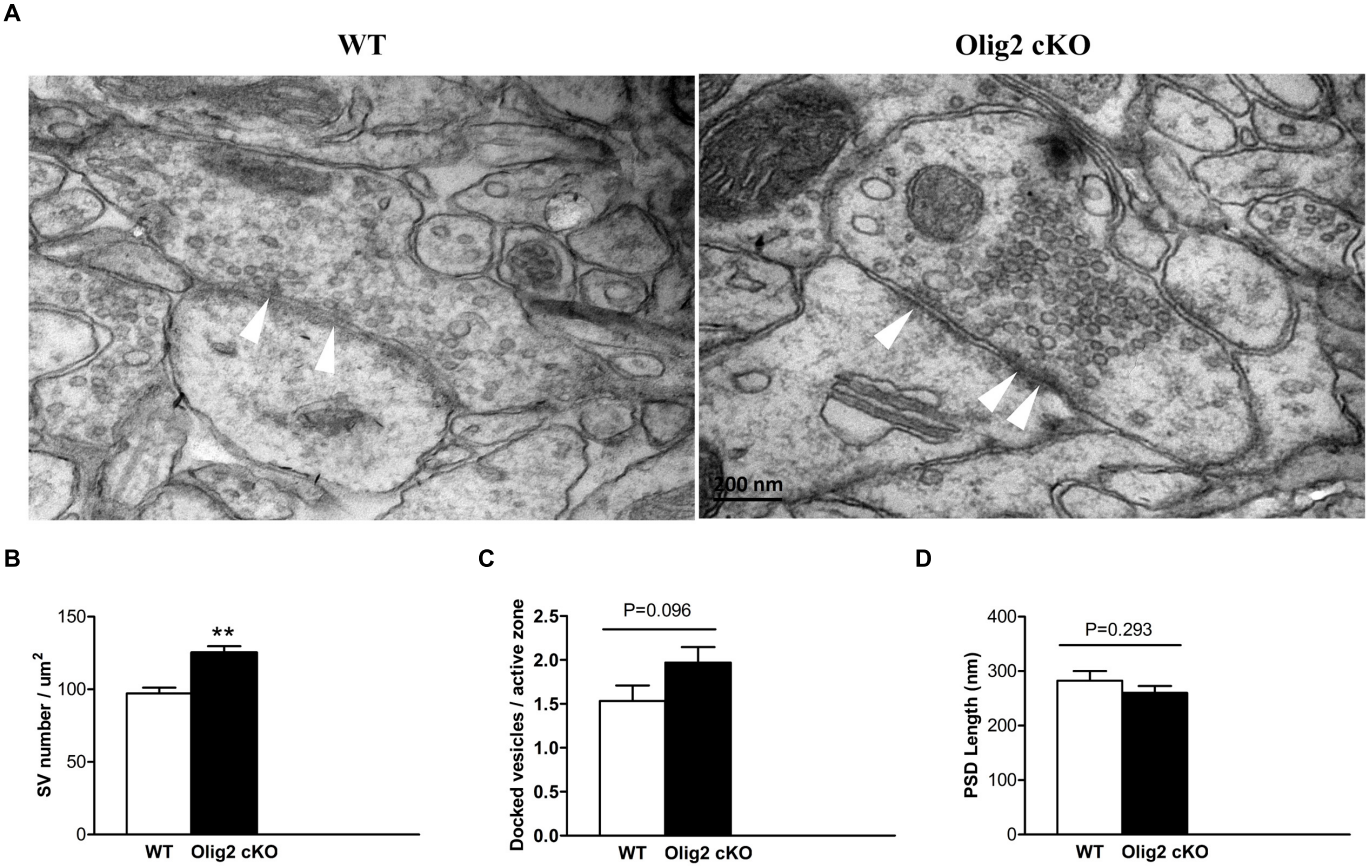
**Electronmicrocopy analysis of glutamatergic synapses and synaptic vesicles in the cortex of WT and *Olig2* cKO mice. (A)** Representative electron microscopy micrographs show an excitatory synapse in the WT and *Olig2* cKO cortices. White arrows indicate docked vesicles. **(B)** Quantification of the density of synaptic vesicles in cortical glutamatergic presynaptic boutons from EM images of WT or *Olig2* cKO mice. **(C)** Quantification of the number of docked vesicles per active zone in WT or *Olig2* cKO excitatory synapses. **(D)** Quantification of the length of the postsynaptic density (PSD) in WT or *Olig2* cKO excitatory synapses. Scale bar = 200 nm, Data are given as mean ± SEM, ^∗∗^*P* < 0.01.

Glutamate is the primary excitatory neurotransmitter, which is largely stored in synaptic vesicles in pyramidal neurons and released upon synaptic stimulation in the cortex (Fonnum, 1984). The long axonal projections of cortical glutamatergic neurons are the primary target for myelination (Tomassy et al., 2014). Moreover, most non-myelinating cortical OLs form contacts with the soma of glutamatergic neurons (Takasaki et al., 2010). Thus, we next questioned whether OL impairment caused by the loss of *Olig2* may affect glutamatergic synapses. Transmission EM images were captured in randomly selected sections and subjected to double blind analysis. The vesicles in each asymmetric synapse with a prominent postsynaptic density (PSD), which is a hall mark of excitatory glutamatergic synapses, were counted in all EM images. Consistent with the increase of overall glutamate levels in the cortex of *Olig2* cKO mice (**Figure 3**), the density of synaptic vesicles within the excitatory presynaptic boutons in the cotex of *Olig2* cKO mice was significantly increased as compared to WT controls (97.2 ± 4.1 in WT vs. 125.2 ± 4.5 in *Olig2* cKO) (**Figures 4A,B**). We also observed a trend of increase in the numbers of docked vesicles at the active zone of *Olig2* cKO boutons (1.53 ± 0.18 in WT vs. 1.97 ± 0.18 in *Olig2* cKO, *P* = 0.096) (**Figure 4C**). No significant difference was detected in the length of PSD between WT and *Olig2* cKO (282.5 ± 17.7 in WT vs. 260.2 ± 12.5 in *Olig2* cKO, *P* = 0.293) (**Figure 4D**). These results suggest that OL impairment caused by the loss of *Olig2* may lead to aberrantly increased glutamate vesicles within synapses and likely increased glutamate release upon synaptic activation.

### *Olig2* cKO Mice Displayed Anxiety-like Behaviors and Deficits in Behavioral Inhibition

**FIGURE 5:**
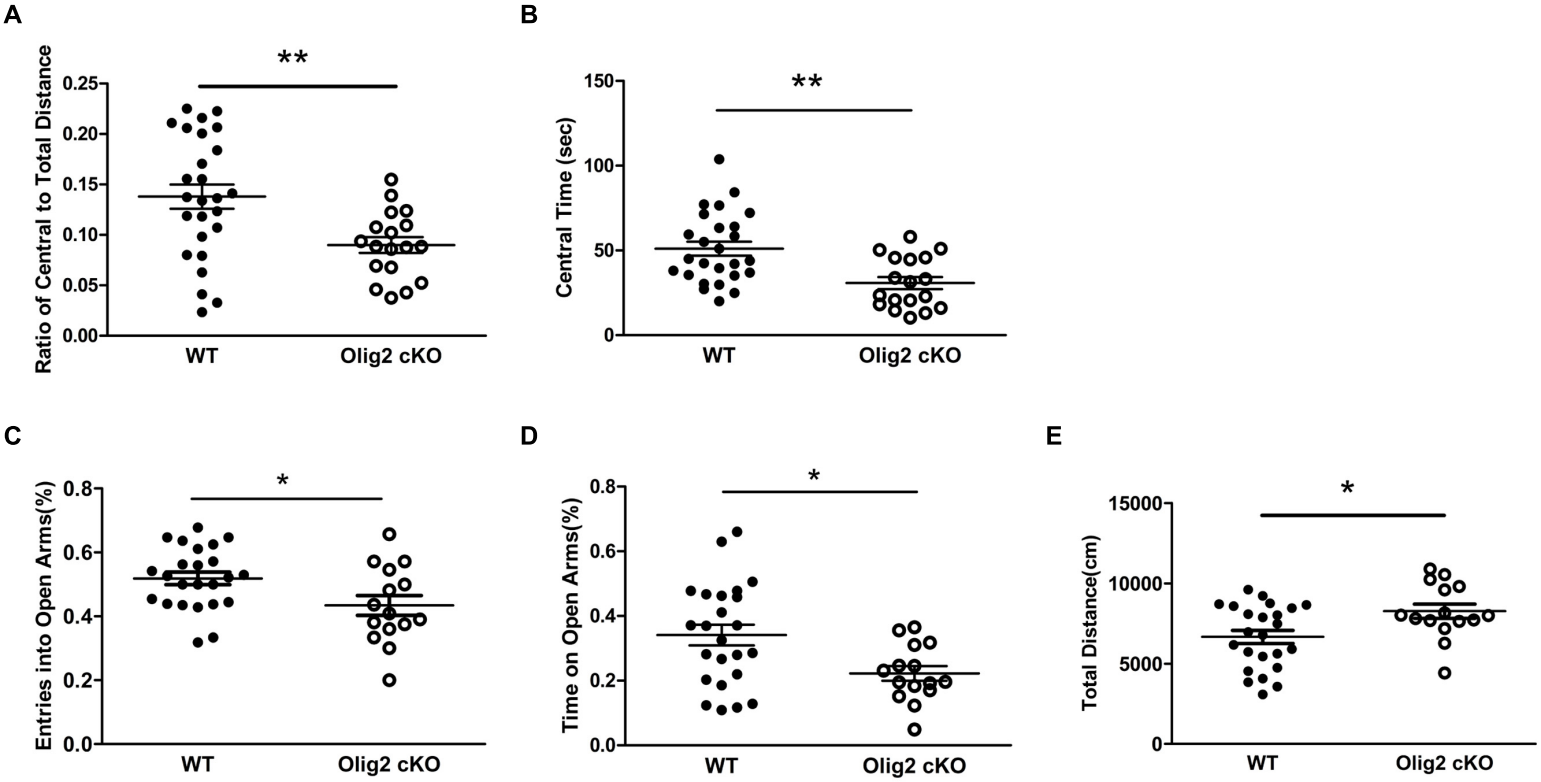
**Anxiety-like behavior tests on WT and *Olig2* cKO mice.** Open field test **(A,B)**: Graphs showed ratio of central to total distance **(A)** and center time **(B)** in WT and *Olig2* cKO mice. Elevated plus maze test **(C–E)**: Graphs showed number of entries into open arms **(C)**, time spent on the open arms **(D)** and total distance traveled on the arms **(E)**, Data are given as mean ± SEM. ^∗^*P* < 0.05, ^∗∗^*P* < 0.01.

We next tested whether OL impairment and glutamate abnormalities may lead to maladaptive behaviors in juvenile mice. We first chose the open field test and elevated plus maze test, which are non-conditioned procedures commonly used for assessing anxiety-like behaviors in rodents (Bourin et al., 2007). In the open field test, juvenile *Olig2* cKO mice showed a significantly lower ratio of travel distance within central area to total travel distance, as compared with WT controls (13.8 ± 1.2% in WT vs. 9.0 ± 0.8% in *Olig2* cKO, *P* = 0.004) (**Figure 5A**) and spent more time in the central area (30.8 ± 3.6 sec in WT vs. 51.1 ± 4.1 sec in *Olig2* cKO, *P* = 0.001) (**Figure 5B**) during the 10-min test, indicating an anxious phenotype. In addition, in the elevated plus maze test, the numbers of entry into the open arms and time spent on the open arms were previously shown to be inversely related with the anxiety level (Xu et al., 2014), hence used to identify anxiety-like behaviors (Brunner et al., 2014). The time spent on the open arms and entries into the open arms were expressed as a percentage of the total time on any arms and entries into any arms during the test. We found that *Olig2* cKO mice showed less entries into the open arms (51.9 ± 2.0% in WT vs. 43.4 ± 3.1% in *Olig2* cKO, *P* = 0.021) (**Figure 5C**) and spent less time on open arms (34.1 ± 3.2% in WT vs. 22.2 ± 2.3% in *Olig2* cKO, *P* = 0.011) (**Figure 5D**), again indicating a more anxious behavior. We also assessed locomotor activity of the animals on the maze by the total distance traveled on both open and closed arms. We found that *Olig2* cKO mice traveled a significantly longer distance than WT controls (6663 ± 404.5 cm in WT vs. 8264 ± 444.3 cm in *Olig2* cKO, *P* = 0.014) (**Figure 5E**), suggesting a higher locomotor activity of *Olig2* cKO mice on the maze. Together, all the aforementioned behavior tests consistently demonstrated anxiety-like behaviors in juvenile *Olig2* cKO mice.

**FIGURE 6:**
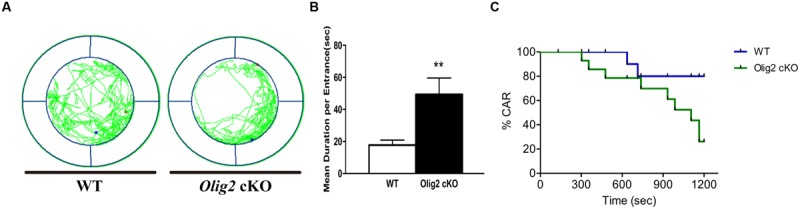
**Impulsivity-like behavior analyses on WT and *Olig2* cKO mice.** Cliff avoidance reaction test **(A–C)**: an example of the travel pathway of WT and *Olig2* cKO on the elevated platform **(A)**. *Olig2* cKO mice failed to avoid the cliff actively by spending significantly more time around the cliff **(B)**. Graphs represent the time course of CAR measurement in WT and *Olig2* cKO mice **(C)**. Data are given as mean ± SEM. ^∗∗^*P* < 0.01.

Impulsivity is defined as a failure in controlling and inhibiting the emotion for appropriate actions and behaviors (Dalley et al., 2008), which is also a typical tendency in adolescence. Recent studies suggest that certain types of functional impulsivity may be linked with anxiety (Taylor et al., 2008). Thus, we explored whether the loss of *Olig2* may also lead to impulsivity-like behavior in juvenile mice. CAR impairment is thought to represent impulsivity-like behaviors in rodents (Matsuoka et al., 2005). Unlike many classical mouse impulsivity tests that require lengthy training, the CAR test can be readily applied to juvenile mice. We found *Olig2* cKO mice traveled more and spent longer time than WT control in the edge area in the CAR test (17.7 ± 3.1 s in WT vs. 49.4 ± 10.2 s in *Olig2* cKO, *P* = 0.007) (**Figures 6A,B**). Moreover, 60% of *Olig2* cKO mice failed to avoid a potential fall from a height, whereas only 14.3% of WT controls fell (**Figure 6C**). These results suggest that the defects in OL maturation due to the loss of *Olig2* also lead to impulsivity-like behaviors.

## Discussion

Using the *Olig2* cKO mouse model, our studies demonstrated that impaired OL maturation can affect glutamate levels in the cerebral cortex and synaptic vesicle density in glutamatergic presynaptic boutons. Furthermore, *Olig2* cKO mice display anxiety-related maladaptive behaviors. To our knowledge, it is the first evidence that demonstrates behavioral abnormalities of *Olig2* cKO mice. These results suggests that OL-neuron interaction in the cortex plays important roles in governing synaptic function and raises an intriguing possibility that impaired *de novo* OL maturation in adolescence may contribute to the early onset of anxiety.

Proton magnetic resonance spectroscopy (1H MRS) is a non-invasive technique that provides great advantages in studying biochemical concentrations of neurotransmitters *in vivo* (Soares and Law, 2009). Specifically, glutamate, which is measured by 1H MRS, has been suggested as an index of cortical excitability (Stagg et al., 2009, 2011). In multiple clinical MRS studies, increased cortical glutamate levels were positively correlated with anxiety symptoms in patients (Phan et al., 2005; Modi et al., 2014). These observations suggest that hyperfunction of cortical glutamatergic neurons may be an important contributing factor for the etiology of anxiety and related disorders. Extensive studies have demonstrated that a wide variety of molecular and cellular mechanisms in neurons and nearby glia cells tightly control glutamate homeostasis and synaptic release (Deitmer et al., 2003; DeSilva et al., 2009). However, which mechanism(s) is dysregulated that underlie the increased glutamate in the cortex of anxiety disorder patients still remains elusive.

Cortical OL maturation occurs concurrently with the most frequent onset of anxiety disorders (Kessler et al., 2005; Miller et al., 2012; Young et al., 2013). In addition, OL and myelin development overlaps with the time for peak binding of cortical glutamate to NMDA receptors in early adolescence (Kornhuber et al., 1988). Therefore, we explored whether selective impairment of OL maturation may affect glutamatergic neurons and behavior in juvenile mice. The *CNP-Cre* mouse line was used in previous studies for specific deletion of genes in mature OL (Wahl et al., 2014; Zou et al., 2014). We employed this mouse line for conditionally deleting the *Olig2* gene that encodes a transcription factor critical for OL development (Zhou et al., 2000; Lu et al., 2002; Ligon et al., 2006; Yue et al., 2006; Mei et al., 2013). Interestingly, *Olig2* cKO mice displayed preferential reduction of cortical mature OLs at P21, whereas OLs in the corpus callosum were much less severely affected. Hence, despite the undefined mechanism that underlies the preferential impairment of OL maturation in the cortex of *Olig2* cKO mouse, this genetic model provides a reasonable tool for dissecting the role of OL maturation in modulating cortical neuronal function during juvenile age. Importantly, using 1H MRS, we detected an overall increase of glutamate in the cortex of *Olig2* cKO mice, suggesting elevated cortical excitability. In addition, we detected increased vesicles in glutamatergic synapses, which further supported glutamatergic hyperfunction as a result of defects in OL maturation. Moreover, juvenile *Olig2* cKO mice displayed anxiety-related behaviors. Together, these observations suggest that normal OL development plays essential roles in governing glutamate signaling and adolescence behavior, and defects in *de novo* OL maturation in early postnatal life could cause aberrant glutamate function and maladaptive behaviors.

Oligodendroglia impairment may affect cortical neurons through multiple distinct mechanisms that still remain elusive. Because the loss of *Olig2* could affect myelination and axonal conductance (Baumann and Pham-Dinh, 2001), neurons in *Olig2* cKO cortex may increase synaptic vesicles as a compensatory adaptation. This may especially affect glutamatergic neurons, because their axons are mostly myelinated by OLs (Tomassy et al., 2014). However, several lines of evidence argue that myelination defects alone do not necessarily cause glutamatergic hyperfunction. For instance, cortical glutamate levels were maintained normal in the heterozygous shiverer mutant mouse that lacked one copy of the *MBP* gene thus displaying hypomyelination without affecting mature OL density (Takanashi et al., 2014). Thus, the preferential impairment of *de novo* OL maturation in the cortex may be a specific mechanism that underlies the aberrant increase of cortical glutamate.

Unlike white matter OLs that perform primary roles in myelin formation, cortical OLs display a radial gradient distribution and differentially myelinate the long-range projections of glutamatergic pyramidal neurons in distinct cortical layers (Tomassy et al., 2014). Besides myelination, the majority of mature OLs reside in deep layers of cerebral cortex are attached with the soma of pyramidal neurons, rather than GABAergic neurons (Takasaki et al., 2010). Although the function of these perineuronal OLs in the cortex still remains unknown, accumulating evidence suggest that OLs can provide metabolic and neurotrophic support (Du and Dreyfus, 2002). In addition, OLs are known to express several glutamate transporters (Domercq et al., 1999; Karadottir et al., 2005), and can uptake glutamate in the gray matter of the developing brain (DeSilva et al., 2009). More importantly, cortical perineuronal oligodendroglia, but not white matter oligodendroglia, express glutamine synthetase (GS) for converting glutamate to glutamine (Damelio et al., 1990). It is well established that glutamine produced by astrocyte GS from the up-taken glutamate can be recycled into neurons, which plays key roles in controlling glutamate recycling (Deitmer et al., 2003). In this regard, cortical gray matter OLs may play important roles in controlling glutamate homeostasis, in parallel with the known function of astrocytes in modulating glutamatergic function.

It is also important to note that besides aberrant glutamate, abnormalities in other neurotransmitters, including GABA, dopamine, serotonin and norepinephrine, are also thought to contribute to anxiety (Kent et al., 2002). Because our understanding of the functional interplay between OLs and neurons is still at its infancy, the types of neurons affected by OL defects still remain undefined. It is conceivable that OL defects in *Olig2* cKO mice could also affect the function of other neuronal cell types in addition to glutamatergic pyramidal neurons, which could contribute to the anxiety-like behaviors we observed in this study. In fact, dopamine transporter knockout mice are severely impaired in CAR test (Yamashita et al., 2013). In addition, disturbing neuregulin-1-ErbB4 signaling in OLs can also lead to increased dopamine receptors and transporters and anxiety-like behaviors in mice (Roy et al., 2007). Whether and how OL maturation may modulate dopaminergic function is a challenging question for future studies. Moreover, the symptoms of anxiety are shared by some other psychiatric disorders, like schizophrenia (Buckley et al., 2009), suggesting that the anxiety like behavior found in Olig2 cKO mice may also contribute to other psychiatric disorders. In fact, ErbB signaling pathway has been considered as a potential therapeutic target for schizophrenia (Deng et al., 2013), which conceivably modulates OL function. However, many well-established behavioral tests for cognitive abnormalities require lengthy training hence could not be applied to juvenile mice. This limits our ability in the current study that aims to identify juvenile abnormalities caused by defects of OL maturation. Future studies may need more specific behavior tests utilized in both young and adult mice to explore whether OL maturation affects higher cognitive function.

## Conclusion

Our studies demonstrate that impaired OL maturation by conditional deleting the *Olig2* gene in differentiated OL can aberrantly increase cortical glutamate, which likely leads to glutamatergic hyperfunction. Moreover, the defects in *de novo* OL maturation clearly result in anxiety-like behaviors and deficits of behavioral inhibition in juvenile mice. Our findings prompt future studies to investigate the intriguing possibility whether defects in OL and/or myelin development in childhood may contribute to the pathogenesis of anxiety in humans.

## Author Contributions

XC, LX, and YF designed the study. XC, WZ and YG acquired and analyzed the data. TL, YT and SL also acquired the data. H-YS analyzed the data. XC, YF and LX wrote the article, which all other authors reviewed. All authors approved the final version for publication.

## Conflict of Interest Statement

The authors declare that the research was conducted in the absence of any commercial or financial relationships that could be construed as a potential conflict of interest.
